# Progression of vertebral deformity of prevalent vertebral fractures in the elderly: a population-based study

**DOI:** 10.1186/s12891-024-07254-y

**Published:** 2024-02-05

**Authors:** Koji Akeda, Kazuma Nakase, Junichi Yamada, Norihiko Takegami, Tatsuhiko Fujiwara, Akihiro Sudo

**Affiliations:** Department of Orthopaedic Surgery, Mie University Graduate School of Medicine, 2-174 Edobashi, Tsu, Mie 514-8507 Japan

**Keywords:** Vertebral fracture, Vertebral deformity, Morphometric fracture, Population-based study

## Abstract

**Background:**

Little is known about the progression pattern of vertebral deformities in elderly patients with prevalent vertebral fractures. This population-based cohort study investigated the incidence, progression pattern, and risk factors of vertebral deformity in prevalent vertebral fractures over a finite period of four years in a population-based cohort study.

**Methods:**

A total of 224 inhabitants of a typical mountain village underwent medical examinations every second year from 1997 to 2009, and each participant was followed up for four years. The extent (mild, moderate, severe) and type (wedge, biconcave, crush) of prevalent vertebral fractures on spinal radiographs were evaluated using the Genant semi-quantitative method. Of these participants, 116 with prevalent vertebral fractures at baseline (32 men and 84 women; mean age: 70.0 years) were included in this study. The progression patterns of the 187 vertebral fractures with mild and moderate deformities (except severe deformity) were evaluated. Logistic regression analysis was used to identify the risk factors associated with deformity progression.

**Results:**

The progression of vertebral deformities was identified in 13.4% (25 vertebral fractures) of the total 187 prevalent (mild and moderate) vertebral fracture deformities over four years. Among the three deformity types, the prevalence of deformity progression was significantly lower in wedge-type vertebral fractures (*P* < 0.05). Age and number of prevalent vertebral fractures per participant were independent risk factors associated with the progression of prevalent vertebral deformities.

**Conclusion:**

This study clarified the natural history of the progression pattern of vertebral deformities in radiographic prevalent vertebral fractures in elderly individuals. Multiple vertebral fractures in the elderly present a risk for the progression of vertebral deformities.

## Background

The occurrence of vertebral fractures (VFs) in the elderly, a majority of which are osteoporotic VFs (OVFs), has a significant impact on the quality of life (QOL) and is associated with an increased risk of disability and mortality [1–3].

The treatment outcomes of VFs in the elderly are usually good; however, major complications, including pseudarthrosis, vertebral collapse, and kyphotic deformity, are challenges observed in the clinical setting [4]. Among these, a kyphotic deformity resulting from VFs [5] is associated with chronic back pain and deterioration of activities of daily living (ADL) and QOL [6, 7].

Previous studies indicated that radiographic prevalent VFs and their numbers have a significant impact on the occurrence of subsequent VFs [8, 9]. However, little is known about the progression pattern of the vertebral deformity of the prevalent VFs themselves; this may influence the progression of the kyphotic deformity. To the best of our knowledge, only one study by Wang et al. [10] evaluated the progression of osteoporotic vertebral deformities in 1533 elderly Chinese female patients over four years. Furthermore, the study showed that prevalent VFs with endplate injuries present a risk for the progression of vertebral deformities. Therefore, we retrospectively investigated the prevalent VFs radiographically in participants of a population-based cohort study using Genant’s semi-quantitative method [11].

This novel study examined the incidence and progression pattern of vertebral deformities in prevalent VFs for a finite period of four years and identified the risk factors for deformity progression in a population-based cohort study.

## Methods

### Participants

This study was approved by the Committee for the Ethics of Human Research of Mie University (IRB reference number: U2018-022) and was performed in accordance with the Declaration of Helsinki. All the participants provided written informed consent.

Data were obtained from the participants of the Miyagawa Study from 1997 to 2009. The Miyagawa study is a population-based cohort study conducted to identify the factors associated with knee osteoarthritis [12], osteoporosis [13], VF [2], and disc degeneration [14] by collecting data from a representative sample of a local elderly Japanese population every second year. Participants aged > 50 years were recruited by invitation to undergo a medical examination from the inhabitants of Odai-cho, a mountain village located in the center of Mie Prefecture (Japan).

Among the seven surveys conducted from 1997 to 2009, 225 participants (68 men, 157 women, mean age: 70.1 years-old) participated in three consecutive surveys (baseline, two and four years).

The participants completed an interviewer-administered questionnaire that included information on age, sex, and the presence of low back pain. Anthropometric measurements included body height, weight, body mass index (BMI: weight [kg]/height^2^ [m^2^]), and bone mineral density (BMD). BMD of the forearm was measured using dual-energy X-ray absorptiometry (DCS-600EX, Aloka, Tokyo, Japan).

## Radiographic assessment of vertebral fractures

Lateral thoracic and lumbar spine radiographs were obtained for each participant. The radiographs were evaluated by a single spine surgeon. The extent (G1, mild; G2, moderate; G3, severe) and type (wedge, biconcave, crush) of prevalent fractures from T4 to L4 at baseline and in the second and final examinations were evaluated using Genant’s semiquantitative (SQ) method [11]. The spinal levels were divided into three groups: thoracic (T4-T9), thoracolumbar (T10-L2), and lumbar (L3-L4). Prevalent VFs were defined as VFs identified at baseline. “Incident VFs” were defined as new-onset VFs found during the second or final examinations that were not identified at baseline.

To assess the intra- and inter-observer reliability of the SQ grading, 27 randomly isolated radiographs were assessed by the same evaluator again after two weeks and by another spine surgeon who was blinded to the SQ grading results. The percentages of agreement for intra- and inter-observer reliability were 98.3% and 98.0%, and the kappa statistics for these were 0.76 and 0.71, respectively.

### Evaluation of the progression in vertebral deformity

G1 and G2 deformities found on thoracic and/or lumbar radiographs at baseline were followed up at two and four years, respectively. Prevalent VFs that changed ‘grade’ or ‘type’ during two or four years compared with baseline classifications were identified as the ‘changed’ group, and prevalent VFs without changes in both ‘grade’ and ‘type’ classifications were identified as the ‘no change’ group. Participants with only a single prevalent VF with G3 deformity at baseline (*n* = 1) were excluded.

### Statistical analyses

Differences in age, body height, body weight, BMI, BMD (young adult mean [YAM]), and VFs numbers between the groups were assessed for statistical significance using an unpaired *t*-test. Differences in the sex ratio, the occurrence of incident VFs between the groups, and the association of progression and deformity type or a spinal level were statistically assessed using the chi-square test, followed by post hoc multiple comparisons using the Bonferroni method, as previously reported [15]. Data are expressed as mean ± standard deviation (SD). Statistical significance was defined as *p* < 0.05.

Logistic regression analysis was used to identify the risk factors associated with deformity progression. Potential risk factors including age, sex, type of deformity, vertebral level, BMD, occurrence of incident VFs, and number of prevalent VFs per participant were assessed. All statistical analyses were performed using IBM SPSS Statistics software (IBM Japan, Tokyo, or IBM Corp., Armonk, NY, USA).

## Results

### Participant characteristics

The characteristics of the 224 participants are summarized in Table 1. Among these, 116 participants (51.8% of the total) had prevalent VFs. There were no significant differences in sex, age, body height, or BMD (YAM) between the VF- and VF + groups. Body weight and BMI were significantly higher in the VF + group than in the VF- group (*P* < 0.05, *P* < 0.01, respectively).

**Table 1:** Characteristics of participants with or without vertebral fracture

	Total	VF-	VF+	P-value
Male	68	36 (52.9)	32 (47.1)	0.35
Female	156	72 (46.2)	84 (53.8)	
Total	224	108 (48.2)	116 (51.8)	
Age (year)	70.1 ± 4.7	70.2 ± 5.1	70.0 ± 4.3	0.77
Height (cm)	149.7 ± 12.4	148.8 ± 16.2	150.5 ± 7.2	0.31
Weight (kg)	53.3 ± 8.0	52.0 ± 8.1	54.5 ± 7.7	< 0.05
BMI (kg/m^2^)	23.5 ± 2.9	23.0 ± 2.7	24.1 ± 3.0	< 0.01
BMD (%)	80.8 ± 14.6	81.1 ± 14.8	80.6 ± 14.4	0.78

VF: vertebral fracture; VF-: participants without a prevalent vertebral fracture; VF+: participants having more than one vertebral fracture; BMI: body mass index; BMD: bone mineral density. BMD is expressed as young adult mean (YAM) values

### Characteristics of vertebral deformity

A total of 200 prevalent VFs were identified at the baseline. Prevalent VFs with G1 grade deformities were observed in 170 vertebrae, those with G2 grade deformities were found in 17 vertebrae, and G3 grade deformities were discovered in 13 vertebrae (Table 2). The prevalence of VFs was highest at the thoracolumbar level (61.0%) for both G1 (61.2%) and G2 (70.6%) grade deformities, followed by thoracic (24.0%) and lumbar (15.0%) levels (Table 2). An equal prevalence of G3 grade deformities was observed at thoracic (46.2%) and thoracolumbar (46.2%) levels. There were no significant differences in the incidences of G1, G2, or G3 deformities at the spinal level (*P* = 0.22).

The prevalence of wedge-type deformity (73.0%) was the highest, followed by biconcave type (18.0%) and crush type (9.0%) (Table 2). Additionally, this trend was detected in G1-, G2, and G3 grade deformities. No significant differences in the incidence of G1, G2, or G3 deformities were observed among the three deformity types (*P* = 0.23).

**Table 2:** Baseline characteristics of vertebral deformity

Deformity Grade	Number of VFs	Spinal level			Deformity type		
		Thoracic	Thoracolumbar	Lumbar	Wedge	Biconcave	Crush
G1	170 (85)	40 (23.5)	104 (61.2)	26 (15.3)	126 (74.1)	29 (17.1)	15 (8.8)
G2	17 (8.5)	2 (11.8)	12 (70.6)	3 (17.6)	9 (52.9)	5 (29.4)	3 (17.6)
G3	13 (6.6)	6 (46.2)	6 (46.2)	1 (7.7)	11 (84.6)	2 (15.4)	0 (0)
Total	200	48 (24.0)	122 (61.0)	30 (15.0)	146 (73.0)	36 (18.0)	18 (9.0)

The extent (G1: mild, G2: moderate, G3: severe) and type (wedge, biconcave, crush) of prevalent vertebral fractures (VFs) were evaluated using Genant’s semi-quantitative method [11]. The spinal levels were divided into three groups: thoracic (T4-T9), thoracolumbar (T10-L2), and lumbar (L3-L4). The numbers in parentheses indicate percentages

### Change in vertebral deformity

Changes in vertebral deformities were identified in 13.4% (25 VFs) of the total of 187 prevalent VFs, including G1 (170 VFs) and G2 (17 VFs) grade deformities.

**Fig. 1:**
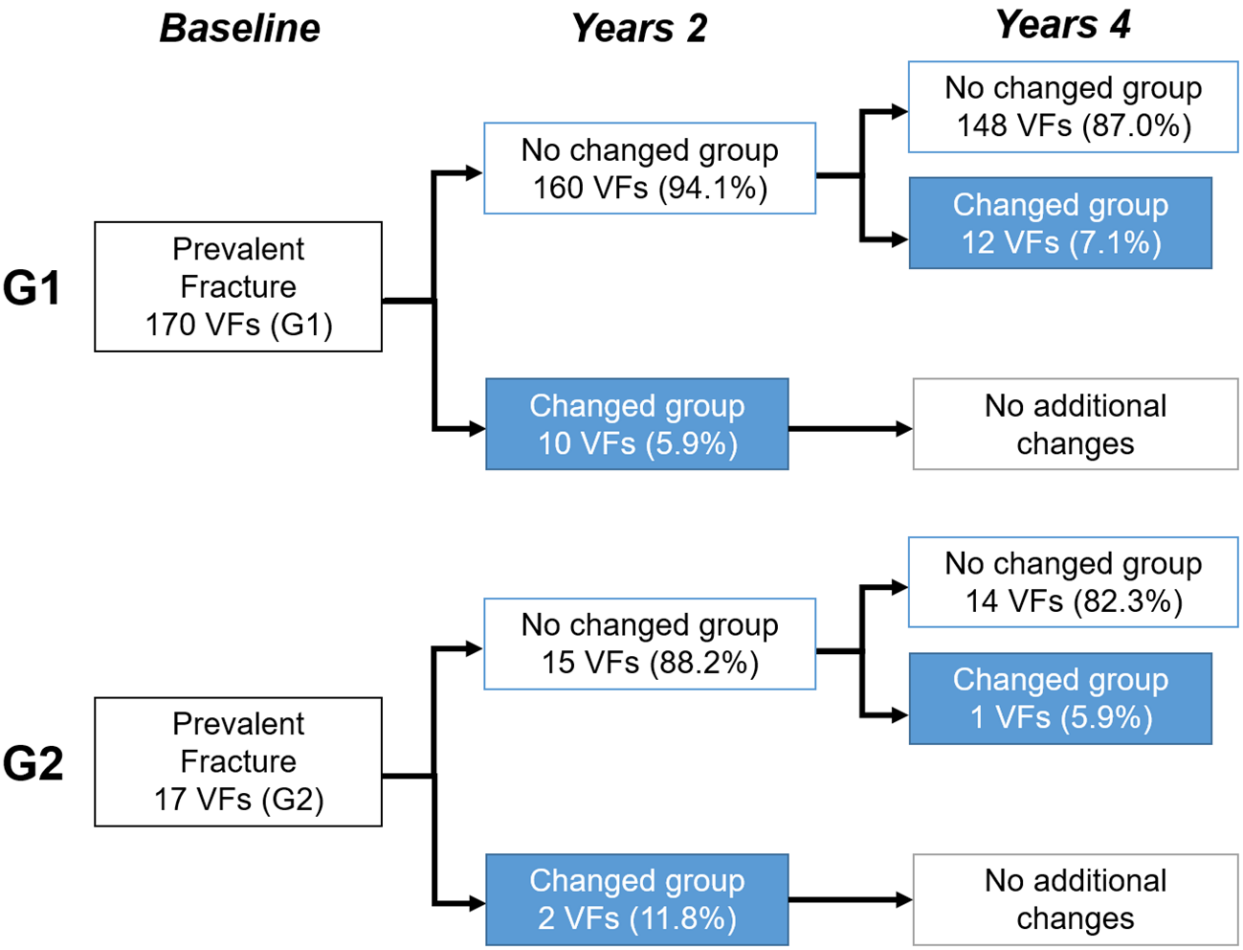
Time-course change in prevalent vertebral fractures (VFs). Prevalent VFs with Genant classification [11] of mild (G1) and moderate (G2) deformities were followed-up for four years

The progression of G1 grade deformities was identified in 22 vertebrae (12.9% of the total; wedge type: 12, biconcave type: 7, crush type: 3) during the four-year observation period. Among these, 10 vertebrae (5.9%) were identified from baseline to second examination, and 12 vertebrae (7.1%) from the second examination to the final examination (Fig. 1). Twelve wedge type G1 grade deformities (W1) progressed deformity grade (W2: 11; W3: 1) (Table 3). In contrast, seven biconcave-type G1 grade deformities (B1) progressed to deformity grade in five VFs (B2: 3; B3: 2) and changed deformity type in two VFs (W2: 1; C1: 1) (Table 3). Three crush-type G1 deformities progressed to deformity grade (C2: 3 VF) (Table 3).

Seventeen prevalent VFs with G2 grade deformity were identified at baseline. Changes in G2 grade deformities were found in 3 vertebrae (17.6%) in this study. Two vertebrae (11.8%) were identified from the baseline to the second examination, and one vertebra (5.9%) from the second examination to the final examination (Fig. 1). Two prevalent VFs of biconcave type G2 grade deformity (B2) and one VF of crush type (C2) progressed to deformity grade (B3: 2; C3: 1) (Table 3).

**Table 3:** Change in vertebral deformity during four years

Baseline		Final observation	
Type / Grade	Number of VFs	Type / Grade	Number (%)
W1	12	W2	11 (91.7%)
		W3	1 (8.3%)
B1	7	W2	1 (14.3%)
		B2	3 (42.9%)
		B3	2 (28.6%)
		C1	1 (14.3%)
C1	3	C2	3 (100%)
B2	2	B3	2 (100%)
C2	1	C3	1 (100%)

The type (**W**: wedge, **B**: biconcave, **C**: crush) and extent (**1**: mild, **2**: moderate, **3**: severe) of the prevalent vertebral fractures (VFs) at baseline and final observation were evaluated using Genant’s semi-quantitative method [11]

### Association of deformity change with deformity type or spinal level

**Fig. 2:**
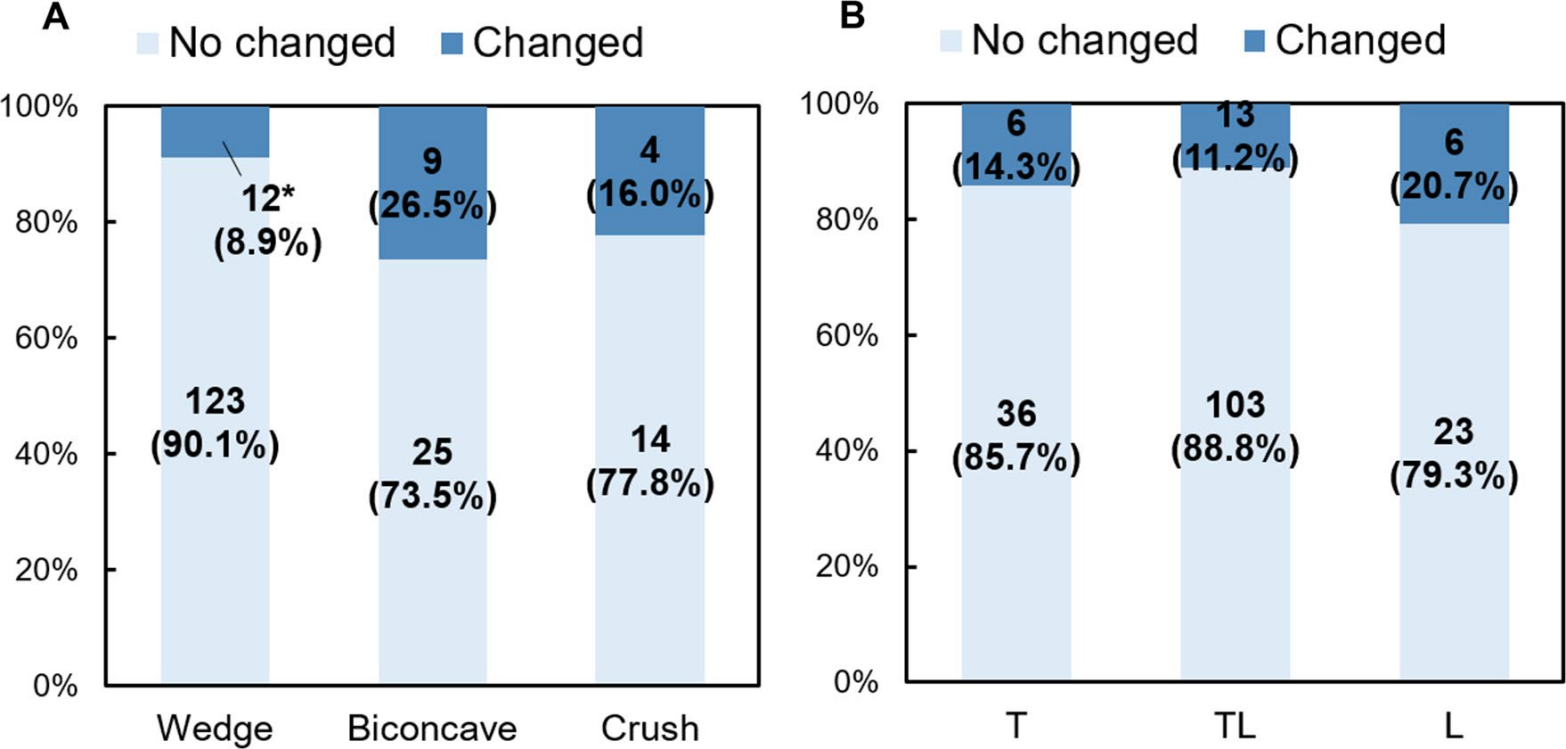
Association between the progression of vertebral deformities and the deformity type (**A**) or spinal level (**B**) *P < 0.05 ( chi-square test). T: Thoracic (T4-T9), TL: thoracolumbar (T10-L2), L: lumbar (L3-L4).

Chi-square tests were conducted to statistically assess whether the deformity type at baseline or spinal level affected the progression of VF deformity over a four-year observation period. There was a significant association between the deformity change and deformity type at baseline (*P* = 0.014). The results of a post-hoc test showed that the number of changed VFs was significantly lower than that expected for wedge-type VFs (*P* < 0.05, Fig. 2A). No significant association was observed between deformity change and spinal level (*P* = 0.40, Fig. 2B).

### Risk factors for deformity change

Among 116 participants who had at least one prevalent VF (G1 and/or G2 grade deformity), 22 participants (19.0%) were included in the ‘changed’ group. The characteristics of both the ‘no change’ and ‘changed’ groups and the results of the univariate analysis between the groups are summarized in Table 4. The age and the number of prevalent VFs per participant were significantly higher in the ‘changed’ group than in the ‘no change’ group (*P* < 0.05, *P* < 0.01, respectively). The prevalence of incident VFs was significantly higher in the ‘changed’ group than that in the ‘no change’ group (*P* < 0.05). No significant differences were found in sex, age, body height, body weight, BMI, BMD, or prevalence of low back pain between the two groups.

**Table 4:** Univariate analysis of baseline characteristics of participants

	Total	‘No change’ group	‘Changed’ group	P value
Prevalent VFs	116	94 (81.0)	22 (19.0)	-
Female, n (%)	84 (72.4)	70 (74.5)	14 (63.6)	0.31
Age (year)	70.0 ± 4.3	69.6 ± 4.0	71.6 ± 5.4	< 0.05
Height (cm)	150.5 ± 7.2	150.5 ± 7.1	150.5 ± 7.9	0.99
Weight (kg)	54.5 ± 7.7	54.6 ± 7.7	54.3 ± 7.8	0.88
BMI (kg/m^2^)	24.1 ± 3.0	24.1 ± 2.8	24.1 ± 4.1	0.96
BMD (%)	80.6 ± 14.4	80.0 ± 14.6	83.1 ± 13.2	0.37
LBP+	65 (56.0)	50 (53.2)	15 (68.2)	0.20
Incident VFs	34 (29.3)	23 (24.5)	11 (50.0)	< 0.05
Prevalent VFs /subject	1.7 ± 0.9	1.6 ± 0.8	2.4 ± 0.8	< 0.01
1 VF/subject	57	53 (56.4%)	4 (18.2%)	< 0.01
2 VFs/subject	37	30 (31.9%)	7 (31.8%)	
≥ 3 VFs/subject	17	11 (11.7%)	8 (50.0%)	

Prevalent vertebral fractures (VFs) that changed grade’ or ‘type’ during the 4-year observation period were classified as the ‘Changed group’, and prevalent VFs without changes in either ‘grade’ or ‘type’ were classified as the ‘no change’ group. BMI: body mass index, BMD: bone mineral density (shown by young adult mean value). The number in parentheses indicates percentage compared to the total number of participants with prevalent VFs. LBP+: participants with low back pain.

Logistic regression analysis revealed that age and number of VFs per participant were significantly associated with the change (progression) in the deformity (Table 5).

**Table 5:** Results of the logistic regression analysis

	B	SE	P value	OR	95% CI for OR
Age	0.13	0.06	0.02	1.14	1.02–1.28
VFs number	1.03	0.30	0.001	2.67	1.49–4.77

CI: confidence interval, OR: odds ratio, SE: standard error, VFs number: number of prevalent vertebral fractures per subject

## Discussion

This population-based cohort study evaluated the incidence and progression patterns of vertebral deformities after VFs over a finite period of four years.

The results of our follow-up study of G1 and G2 deformities revealed that the incidences of progression in vertebral deformity during four years were 12.9% and 17.6%, respectively. One of the pathomechanisms underlying the progression of VF deformities is the refracture of existing VFs. Wang et al. [10] evaluated the progression and incidence of prevalent VFs of osteoporotic vertebral deformities in 1533 Chinese women over four years using Genant’s SQ method [11]. Similar to the results of our study, they stated that 8% of G1 deformities and 10.6% of G2 deformities had progressed to at least one deformity grade.

Another pathomechanism of deformity progression is the progression of vertebral collapse during the healing process of newly incident VFs. Jeon et al. [16] investigated the progression of vertebral deformities in 55 consecutive patients with OVFs who were treated conservatively for a minimum follow-up of six months. They stated that the vertebral deformity ratio (vertebral collapse ratio) had time-dependently increased from 35% at baseline to 63% at six months. Moreover, Okuwaki et al. [17] reported that the mean vertebral deformity ratio (collapse ratio) at six months after injury was 46.1% in 70 postmenopausal women. These previous reports suggest that the vertebral deformity of new incident VFs naturally progressed six months post-injury. However, the possibility of recognizing the early phase (less than six months) of VFs after the injury as prevalent VF at baseline would be extremely low in this population-based study.

The results of our follow-up study revealed that wedge-type deformities progressed to deformity grade over four years; however, biconcave-type deformities changed both deformity type and grade. Additionally, our results disclosed that the prevalence of deformity change was the highest in the biconcave type. Biconcave-type deformity also referred to as ‘codfish vertebra,’ was associated with multilevel VFs in severe osteoporosis [18]. Jones et al. [19] evaluated the relationship between vertebral deformities and BMD and reported that BMD was strongly associated with the occurrence of biconcave-type deformities than wedge- or crush-type deformities. These previous reports suggest that the biconcave type of deformity is susceptible to changes in deformity resulting from mechanical stress applied to the VFs owing to vertebral fragility than other types of deformities.

In this study, no significant differences in BMD measured at the forearm were identified between participants with and without VFs or between the ‘no change’ group and the ‘changed’ group. Previous studies indicated that there is a wide range of differences in BMD depending on the different skeletal sites measured [20]. It has been reported that BMD measured at the lumbar spine or hip exhibited a better correlation with the occurrence of VFs than that measured at the forearm [21]. Therefore, differences in BMD depending on the presence or progression of VFs deformity may be obtained by measuring the BMD in the lumbar spine or hip. A medical history of osteoporosis may affect the progression of VF deformities [17]. However, the treatment rate of osteoporosis of total participants in this cohort was low (7.1% of total participants) and it did not differ significantly between the ‘no change’ and ‘changed’ groups (data not shown).

Logistic regression analysis revealed that age and the number of prevalent VFs per participant were independent risk factors for progressive changes in vertebral deformities. Numerous studies have stated that the presence and/or number of prevalent VFs are independent risk factors for subsequent (incident) VFs [8, 9]. Additionally, the progression of vertebral deformity of prevalent VFs defines the criteria for incident VFs [10, 22, 23], suggesting that the number of prevalent VFs affects not only the occurrence of incident VFs but also the progression of prevalent vertebral deformities by the refracture of existing VFs.

In 1996, Genant et al. [11] reported a semi-quantitative method for diagnosing VFs with excellent intra-observer and good inter-observer agreements. Recent large prospective studies [24, 25] compared the diagnosis and prevalence of osteoporotic VFs using morphometric (quantitative [25] or semiquantitative [24] methods) and morphological (algorithm-based qualitative [ABQ] method [26]) approaches. They reported that the prevalence and occurrence of spinal levels of VFs differed significantly between the two approaches. Lentle et al. [24] stated that VFs obtained using a morphological approach were highly correlated with bone mineral density (BMD), incident VFs, and non-vertebral fractures than those obtained using Genant’s semiquantitative method [11]. A recent review of the diagnosis of osteoporotic VFs [27] suggested that the morphological approach is the preferred strategy for diagnosing osteoporotic VFs from spinal radiographs. Therefore, the prevalence and incidence of VFs should be evaluated using both morphometric and morphological approaches in future studies.

Our study has several limitations. Miyagawa (Odai-cho) is a mountain village with many inhabitants who are engaged in forestry. Therefore, there are potential differences in the occupation ratio compared with that of the general Japanese population. Furthermore, the health condition of the inhabitants, including bone health of the spine, may differ from that of urban dwellers. Hence, a multi-cohort study with diverse demographic characteristics would improve the generalizability of the results. Second, the participants of this study were selected from among the inhabitants who participated in medical surveys conducted between 1997 and 2009. Therefore, the baseline characteristics of the participants, including the incidence of VFs, BMD, and the osteoporosis treatment rate, may differ depending on the year surveyed. In addition, the participants who completed the three continual medical surveys were healthy without serious illness and/or severe physical disability. Third, spinal alignment, especially sagittal balance, was not evaluated using spinal radiography. Since sagittal spinal alignment is a risk factor for VFs [28], the sagittal spinal imbalance caused by multilevel VFs may have affected the progression of VF deformity in this study. Fourth, our sample size was small. A larger sample size is required to increase the reliability of the results. Fifth, the four-year follow-up period was too short to fully capture the natural history of the vertebral deformity. Future long-term follow-up studies are required to comprehensively assess the progression of vertebral deformities over time. Finally, the onset time of prevalent fractures, which may affect the progression of vertebral deformities, is unknown in this study. Therefore, additional image analysis, such as magnetic resonance imaging (MRI), would be needed to identify it in future studies.

## Conclusions

This population-based study revealed that 13.4% of prevalent VFs progressed in deformity type and/or grade over four years. Furthermore, the age and the number of prevalent VFs per participant were independent risk factors for VF deformity type progression. Therefore, the results of our study suggest that in a clinical setting, elderly patients with multiple VFs have a risk of VF deformity progression that may lead to significant changes in spinal alignment.

## Data Availability

The datasets used and analyzed in the current study are available from the corresponding author upon reasonable request.

## Abbreviations

VFs: vertebral fractures
BMI: body mass index
BMI: bone mineral density
